# The influence of emotional face distractors on attentional orienting in Chinese children with autism spectrum disorder

**DOI:** 10.1371/journal.pone.0250998

**Published:** 2021-05-04

**Authors:** Li Zhang, Guoli Yan, Valerie Benson

**Affiliations:** 1 Faculty of Psychology, Tianjin Normal University, Tianjin, P. R. China; 2 School of Psychology, University of Central Lancashire, Preston, United Kingdom; 3 Center of Collaborative Innovation for Assessment and Promotion of Mental Health, Tianjin, P. R. China; Bournemouth University, UNITED KINGDOM

## Abstract

The current study examined how emotional faces impact on attentional control at both involuntary and voluntary levels in children with and without autism spectrum disorder (ASD). A non-face single target was either presented in isolation or synchronously with emotional face distractors namely angry, happy and neutral faces. ASD and typically developing children made more erroneous saccades towards emotional distractors relative to neutral distractors in parafoveal and peripheral conditions. Remote distractor effects were observed on saccade latency in both groups regardless of distractor type, whereby time taken to initiate an eye movement to the target was longest in central distractor conditions, followed by parafoveal and peripheral distractor conditions. The remote distractor effect was greater for angry faces compared to happy faces in the ASD group. Proportions of failed disengagement trials from central distractors, for the first saccade, were higher in the angry distractor condition compared with the other two distractor conditions in ASD, and this effect was absent for the typical group. Eye movement results suggest difficulties in disengaging from fixated angry faces in ASD. Atypical disengagement from angry faces at the voluntary level could have consequences for the development of higher-level socio-communicative skills in ASD.

## Introduction

Autism Spectrum Disorder (ASD) is a lifelong neurodevelopmental condition characterized by social and communicative abnormalities and repeated and stereotyped behaviours [1]. Individuals with ASD have been shown to have significant deficits in social cognition, for example, this population have poorer performance in recognising facial emotions compared to typically developing (TD) individuals, especially for negative (e.g. angry and fearful) emotions [2–5]. Impaired social cognition is regarded to be related to atypical attentional processing of social stimuli [6–8], as abnormal attention to social cues may impede rapid detection and utilisation of key information in the social environment, and thus may impact on the development of normal social and cognitive behaviours in autism [9, 10].

In order to understand the underlying mechanisms of atypical social and cognitive development in ASD, a number of studies have sought to explore the attentional processes related to emotional faces in autism, in which angry faces are particularly utilized as an example of negative expressions. Although a deficiency in attentional orienting has been predicted for emotional faces in ASD, numerous studies fail to detect any obvious group differences. By adopting the face-in-the-crowd task [11], several studies have found a detection superiority for angry faces in both the ASD and TD groups, whereby all participants respond faster to the angry face, which is presented among an array of neutral face distractors, compared to the happy face condition ([12–16], but see also the contrary evidence from [17]). In addition, Yerys et al. [18] reported an advantage of early visual attention processing of angry faces versus neutral faces shown in a rapid serial visual processing stream in ASD. Furthermore, other studies [19–21] that have utilised the spatial cueing paradigm (SCP) [22], have revealed similar performance for covert orienting to peripheral emotional faces presented as valid cues for a short duration of 500ms, to the position of the subsequent target in both ASD and TD groups. These findings suggest that automatic (or involuntary) attentional orienting towards, or early visual processing of, angry faces as well as happy faces is intact in ASD individuals.

However, using a similar SCP paradigm, several studies have also demonstrated evidence of atypical attentional disengagement from negative emotional stimuli in ASD. For example, García-Blanco et al. [23] found that when angry faces were presented as valid location-related cues for 1500ms, the ASD group took longer to respond to the target relative to the TD controls. A similar result has been found by Antezana et al [24], and this effect has been taken as evidence of quick visual disengagement (or attentional inhibition) for threatening stimuli at the voluntary control (or endogenous) level in ASD. However, and in contrast, May et al. [21] and Milosavljevic et al. [25] failed to report any attentional disengagement differences related to emotional faces shown as valid or invalid cues in the SCP in ASD, which is out of line with previous results [23, 24]. Importantly, these divergent results seem to point to an inefficiency of the SCP to measure the specific attentional processes of spatial emotional stimuli cues. Slower responses in the valid angry face cueing condition [23, 24] could simply reflect a delayed motor execution caused by the high arousal from angry faces in ASD, rather than a tendency of quick attentional disengagement from angry faces in ASD [26]. Moreover, without the recording of eye movements to highlight the temporal and spatial information related to attentional processing in the SCP, it is difficult to differentiate between the exogenous orientation and endogenous disengagement processes for spatially presented emotional cues by adopting manual reaction time as the sole dependent measure. In addition to this, other studies have reported increased attention to negative stimuli in ASD. For example, Isomura, Ogawa, Shibasaki & Masataka [27] found that ASD children take longer to detect the target when threatening stimuli (snakes) are shown as distractors, indicating that individuals with ASD could have difficulties in disengaging from different types of negative stimuli. The inconsistencies in the results to date demonstrate that paradigms used in previous studies may be unsuitable in their ability to provide accurate and clear measures of both exogenous and endogenous attentional characteristics for emotional information in autism [23, 24].

Investigating the nature of any differences in attentional processing of emotional faces in ASD will contribute to an understanding of the nature of atypical social processing in this group. The current study aimed to adopt the remote distractor paradigm (RDP) [28] to investigate attentional processing of emotional faces in ASD. By asking participants to make eye movements to a target presented in isolation or with a central, parafoveal and peripheral distractor, the RDP has revealed the influence of non-social visual distractors on both exogenous orienting (saccadic errors made towards to the distractors instead of the target) and on endogenous orienting (saccade latencies or time needed to initiate an eye movement to the target) simultaneously in typical and ASD populations [29]. In the RDP, saccadic errors towards the distractors indicate a complete failure of suppressing involuntary saccade responses, and therefore, this measure reflects the influence of visual distractors on attentional control at the reflexive or exogenous level. In contrast, saccade latencies reflect the time that participants need to disengage from the presented distractors successfully, when they are able to suppress reflexive responses towards the distractors, and make voluntary saccades to the target. As such, the saccade latency measure indicates the influence of distractors on the attentional orienting at the voluntary or endogenous level. Previous studies [30, 31] have also shown that emotional distractors produce increased remote distractor effects in the RDP. These findings suggest that the RDP permits an investigation of the influence of emotional faces at both the exogenous and endogenous levels in ASD and TD children.

In line with previous reports we predicted an intact ability to orient reflexively to emotional face distractors in ASD, and we expected that the proportion of exogenous saccade errors made towards the irrelevant angry and happy face distractors to be higher, compared to the neutral face distractors, in both groups. Secondly, if ASD children perform typically in voluntary attentional processing of emotional information, both groups should take longer to disengage from emotional distractors compared to neutral face distractors. However, if ASD children show atypical disengagement from emotional stimuli, for example, rapid disengagement from the angry faces, we would predict that emotional effects related to angry faces would impact upon disengagement speed such that this would be reduced in the ASD group compared to the TD group. Alternatively, if there is increased delayed disengagement from negative stimuli in ASD, we would predict increased distractor effects for angry faces in the ASD group. This atypical attentional processing, either of faster or slower disengagement, would be especially obvious for the central distractor conditions.

## Methods

### Participants

Fifteen ASD children (2 females and 13 males, Chinese) and 19 typical children (3 females and 16 males, Chinese) aged from 60 to 90 months old were recruited from the kindergartens in Tianjin, China. Parents reported no history of neurodevelopmental damage or delay in all children from the TD group. Prior to the formal study, parents of all participants read and demonstrated understanding of the procedures in the study and signed the informed consent forms. The procedures of the current study were approved by the Ethical Committee of Tianjin Normal University.

Children with ASD were officially diagnosed with an ASD by at least one experienced clinician. All the ASD diagnosis criteria were consistent with the requirements reported in the fifth edition of the Diagnostic and Statistical Manual of Mental Disorders [1]. The Chinese version of the Autism Spectrum Quotient: Children version [32, 33], was adopted to assess autism symptoms of all participants by either parents or teachers and the ASD group scored higher (above the cutoff of 76) on AQ compared to the TD group, *t* = 4.23, *p* < .001 (see Table 1 for details of AQ scores for both groups). This finding on AQ scores validates the original clinical ASD diagnoses.

**Table 1:** Demographic data (mean ± SD) of the ASD and TD groups on age, IQ and AQ scores.

	ASD (n = 15)	TD (n = 19)	*t*-value	*P*
Age(months)	71.67 (8.06)	70.21 (2.27)	0.75	.46
VIQ	111.80 (16.14)	110.21 (8.36)	0.37	.71
PIQ	107.13 (13.03)	109.74 (12.32)	-0.60	.56
FSIQ	110.07 (12.27)	107.47 (9.82)	0.69	.50
AQ	80.33 (11.47)	63.68 (11.01)	4.23	**< .001**

Note: Specific data on socioeconomic status were not collected in the current study.

The Chinese version of the Wechsler Preschool and Primary Scale of Intelligence: Fourth Edition [34] was used to measure participants’ cognitive abilities. Both groups were matched on intelligence quotients (IQ), showing similar scores on verbal (VIQ), performance (PIQ) and full-scale (FSIQ) profiles, |*t*|s < 0.8, *p*s >.40. There were no group differences in chronological age (CA), *t* = 0.75, *p* = .46 (see Table 1 for details of IQ scores and CA for both groups).

### Apparatus

An EyeLink Portable Duo (S.R. Research Ltd, Canada) eye-tracker with a sampling rate of 500 Hz was used to record the eye movement data. Experimental stimuli were displayed on a 19-inch DELL monitor (1024 × 768 pixels resolution). The refresh rate of the display screen was 75 Hz. All participants rested upon a chin rest to maintain head stability during formal testing.

### Materials

**Fig 1:**
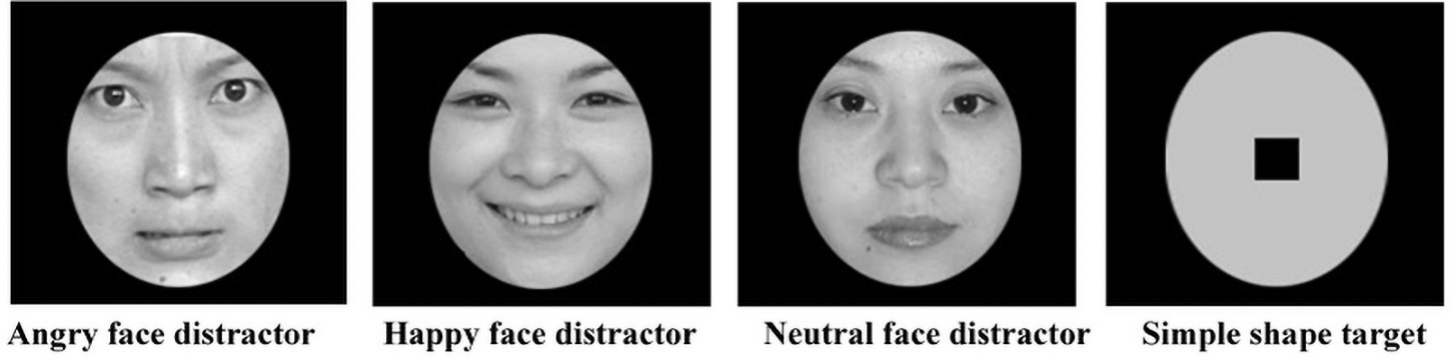
Three categories of emotional face distractor examples and the simple shape target used in the RDP task. The face images were taken from the Chinese Affective Face Picture System (CAFPS, Wang & Luo, 2005, Gong, Huang, Wang, & Luo, 2011), and all the face models in the CAFPS gave their consent for publication for research purposes.

The target was a simple ellipse shape with a central black square. Fifty-four face models with angry, happy or neutral expressions were selected as experimental distractors from the Chinese Affective Face Picture System (CAFPS) [35]. Each expression condition had 8 female and 10 male models. For angry and happy faces, there were 7 models and 6 models with the mouth open. Additionally, six further faces (not used in the formal experimental trials) consisting of two angry, happy or neutral expressions were chosen as practice stimuli. The face models from the CAFPS that we used in the current study all provided written informed consent to publish their images for research purposes [35, 36]. Both the target and distractors were grayscale and were in the same oval template, size 4.35° X 5.42°(135 X 158 pixels). Example stimuli are shown in Fig 1.

Validation data for emotional valence and arousal for the experimental emotional faces was collected based on a 9-point Likert scale measurement, based on the work of Gong et al. [35] and Wang et al. [36], and the data were analysed using the one-way ANOVA method. There was a significant emotion type effect on valence, *F* (2, 51) = 228.51, *p* < .001, and on arousal, *F* (2, 51) = 26.40, *p* < .001. Post-hoc analysis showed that angry faces scored lowest on valence (*M* = 2.50, *SD* = 0.38), with neutral faces (*M* = 4.16, *SD* = 0.34) in the middle rank and happy faces (*M* = 6.25, *SD* = 0.76) showed the highest scores, *p*s < .001. These results confirm the negative valence for angry faces, positive valence for happy faces and middle valence for neutral faces. Arousal scores were higher in angry (*M* = 6.65, *SD* = 1.22) and happy (*M* = 6.39, *SD* = 0.86) faces than neutral (*M* = 4.59, *SD* = 0.59) faces, *p*s < .001, and no difference of arousal was detected between angry and happy faces, *p* = 1.000. Brightness values were also collected in Adobe Photoshop for each face model embedded in the black background with the target. Comparison results showed that brightness values were similar in angry (*M* = 3.72, *SD* = 0.15), happy (*M* = 3.66, *SD* = 0.10) and neutral (*M* = 3.67, *SD* = 0.12) face conditions, *F* (2, 51) = 1.35, *p* = .27.

Three categories of emotional face distractors were blocked into different experimental sessions. In each block, there were 144 trials, including 36 single target trials and 108 distractor trials. Distractor faces were presented at central (central point of the display screen), parafoveal (5° from the centre of the display screen) or peripheral (10° from the centre of the display screen) positions synchronous with the target. Targets were presented on either the right or left side 5° or 10° away from the centre of display screen in the single target and central distractor trials. In parafoveal and peripheral distractor conditions, the target and distractor were located at the mirror opposite location of each other. For each distractor type presented at each distractor position, there were 36 trials. In total, including trials with a single target and trials with both a distractor and a target, each participant was required to complete 432 trials.

### Procedure and eye movement recording

Following an explanation of the instructions to the participants, participants were asked to verbalise the task requirements, or to point out the target to look at and the distractors to be ignored. Participants also completed the RDP saccade procedure presented serially in slides and then received a practice session on the eye tracker to become familiar with the eye movement procedures.

**Fig 2:**
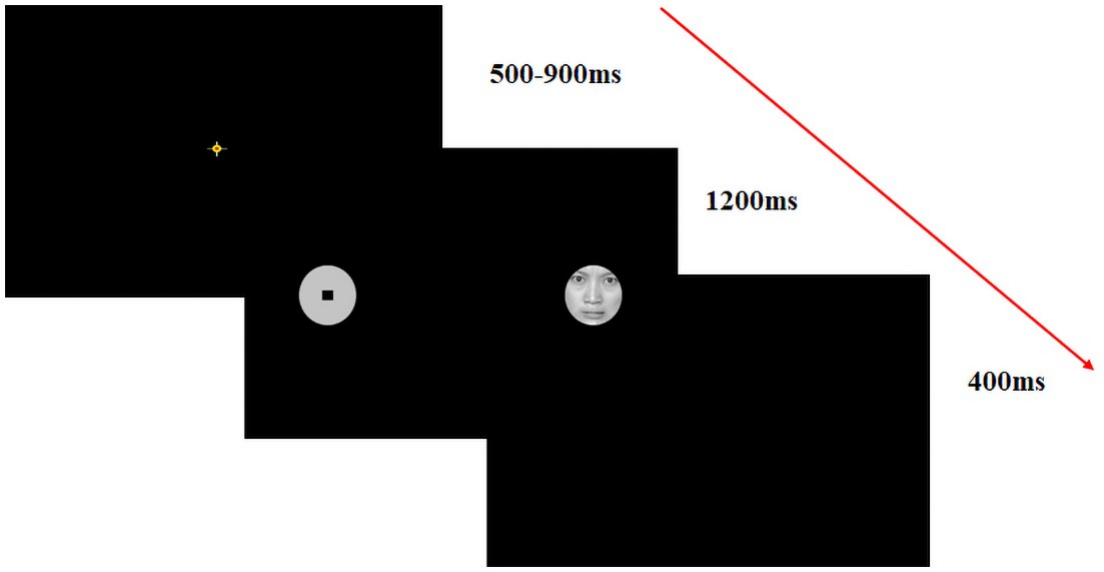
A schematic example of a distractor trial sequence in the RDP whereby an angry face distractor and the target were shown in peripheral vision away from the centre of the display screen.

In the formal testing sessions, participants firstly received a three-point-calibration test, in which fixational positions of the eye at different locations on the display screen were recorded. The calibration test was accepted with an average calibration error below 0.5° for each child. Before each trial participants were required to look at a small point presented at the centre of the display screen, to correct for drifts. Following drift correction each trial began with the presentation of a fixation cross (1°) at the centre of the screen for a variable duration of 500-900ms. Following fixation of the central cross, a target display was presented for 1200ms, and during this period participants were required to ignore any distractors if present, and to look to the centre black square of the target as rapidly and accurately as possible. Finally, a blank screen was presented for 400ms to end the trial sequence (Fig 2 presents a schematic of a trial sequence).

### Eye movement measures

The current study analysed three eye movement measures: saccadic errors (first eye movements executed towards distractors with amplitude greater than 2.2°), saccade latency (for correct trials in which the first saccade was initiated towards the target, and with saccade amplitude greater than 2.2°), and, failure to disengage from the central distractors in the first saccade (with saccade amplitudes less than 2.2°). The selection of the saccade amplitude of 2.2° was based on previous criteria adopted in RDP studies (2°) [29–31], and also based on the size of the current stimuli (4.35° X 5.42°) which ensured that first saccades with an amplitude greater than 2.2° were not reflecting eye fixations within the stimuli. The former two eye movement measures are typically adopted in studies to indicate the effects of irrelevant distractors on both the reflexive orienting system (errors) and the voluntary orienting system (latency). The other measure, disengagement failure rate (DFR), adopted in the current study resulted from the frequent observation of trials in which participants were unable to disengage from centrally presented distractors in the first saccade. Making an eye movement within the distractor face was considered an indicator of disengagement difficulty at the voluntary level in this study.

### Data exclusion criteria and analysis

Consistent with previous RDP studies [29–31], prior to statistical analyses trials were removed according to the following criteria (1) a blink was made during the first saccade (2.66%). (2) start position of the first saccade was beyond 1° from the centre of the screen (7.06%), (3) saccade latency were less than 80ms (anticipatory saccade, 2.10%) [37], (4) amplitude of the first saccade was less than 2.2° in parafoveal, peripheral distractor conditions and single target condition (0.56%), (5) a saccade of more than 2.2° was made towards the opposite direction of the target in single target and central distractor conditions (0.26%), and (6) saccade latencies were greater or lower than 3 standard deviations from mean value of each individual participant (0.58%). A total of 12486 trials were included in the formal analyses.

The Linear mixed models (LMMs, from lme4 package of version 1.1–7) was used to analyse valid data in the R environment (R Development Core) [38]. Group (between-subjects factor), distractor expression (within-subjects factor) and distractor position (within-subjects factor) were fitted as the fixed factors. The maximum random effects structure, including random intercepts and random slopes for fixed effects over both participants and items, were considered when the LMMs could converge. If the maximum model could not been fitted, simple random effects model was adopted as the optimal method according to the likelihood-ratio test result [39]. Log-transformed saccade latency was adopted in the LMMs analysis. Comparison differences between pairwise conditions or interactions were indicated by t-value for saccade latency to reduce the impact of data skewness. Analyses results for error rate and DFR were indicated by z-value by using logit-link function. An absolute value of more than 1.96 for each t or z result was accepted to indicate an observable difference or effect at the 0.05 alpha level.

## Results

### Directional error

Directional error rate was computed by dividing erroneous trials, where participants made the first eye movement towards the distractor instead of the target, by total valid trials in parafoveal and peripheral conditions. Descriptive statistics for error rates and for the other two eye movement measures are shown in Table 2. S1–S3 Tables are presented in the supporting information. S1 Table shows the statistical estimates of the fixed effects for the error rate.

**Table 2:** Means and standard deviations of eye movement measures recorded for neutral, happy and angry face distractors in central (C), parafoveal (NR), peripheral (FAR) and single target (ST) conditions in both groups.

	ASD				TD				
		C	NR	FAR	ST	C	NR	FAR	ST
Neutral face distractors	SL (ms)	297 (97)	252 (69)	232 (71)	186 (55)	323 (118)	270 (79)	246 (78)	213 (83)
	ER		0.49 (0.50)	0.52 (0.50)			0.40 (0.49)	0.46 (0.50)	
	DFR	0.16 (0.36)				0.16 (0.37)			
Happy face distractors	SL (ms)	297 (95)	262 (70)	237 (71)	183 (55)	331 (111)	271 (83)	245 (72)	209 (77)
	ER		0.57 (0.50)	0.59 (0.50)			0.52 (0.50)	0.50 (0.50)	
	DFR	0.13 (0.34)				0.17 (0.37)			
Angry face distractors	SL (ms)	314 (104)	256 (73)	233 (68)	185 (57)	325 (113)	267 (75)	243 (71)	199 (67)
	ER		0. 60 (0.49)	0.59 (0.50)			0.56 (0.50)	0.50 (0.50)	
	DFR	0.24 (0.43)				0.15 (0.36)			

Note: SL refers to the saccade latency; ER to the error rate and DFR to the disengagement failure rate.

**Fig 3:**
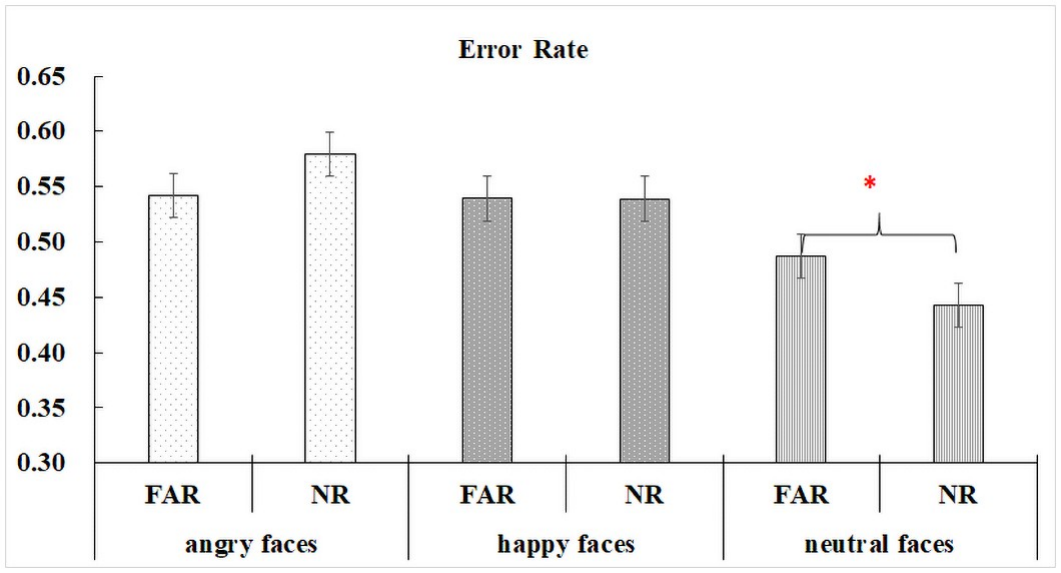
Interaction effects between angry and neutral face distractor conditions on distractor position error rate differences for all participants.

Significant differences among distractor types were observed, whereby error rates were higher in angry (*M* = 0.56, *SD* = 0.50) and happy (*M* = 0.54, *SD* = 0.50) face distractor conditions relative to the neutral (*M* = 0.47, *SD* = 0.50) face distractor condition, |*z*|s > 3.90, *p*s < .001. There was no group or distractor position effect. A significant interaction by distractor position and distractor type (angry faces vs neutral faces) was found, *z* = -2.43, *p* = .015, showing that neutral face distractors triggered more errors in the peripheral (*M* = 0.49, *SD* = 0.50) location compared to the parafoveal (*M* = 0.44, *SD* = 0.50) location, *z* = -2.02, *p* = .043. However, for angry face distractors, error rate differences in peripheral (*M* = 0.54, *SD* = 0.50) and parafoveal (*M* = 0.58, *SD* = 0.49) distractor conditions were non-significant, *z* = 1.73, *p* = .084 (see Fig 3).

The eccentricity effects show that neutral faces presented in the periphery are more difficult to ignore at the involuntary attention level, and thus result in more unexpected eye movements towards them in contrast to parafoveal neutral faces. Similar results have also been reported in previous RDP studies [30]. In contrast, result patterns for emotional faces, in particular angry faces, indicate that the influence of emotional stimuli on reflexive orienting is not modulated by distractor position in young children with and without ASD, and that threatening faces presented within the peripheral visual field have a robust ability to capture visual attention reflexively.

### Saccade latency

Basic distractor effects between single target and distractor trials were firstly compared for each expression block. Saccade latencies were shown to be shorter in the single target condition than in distractor trials in both groups, regardless of emotional distractor type, *|t*|s> 9, *p*s < .001. Group differences and interactions were not significant for this basic distractor effect.

**Fig 4:**
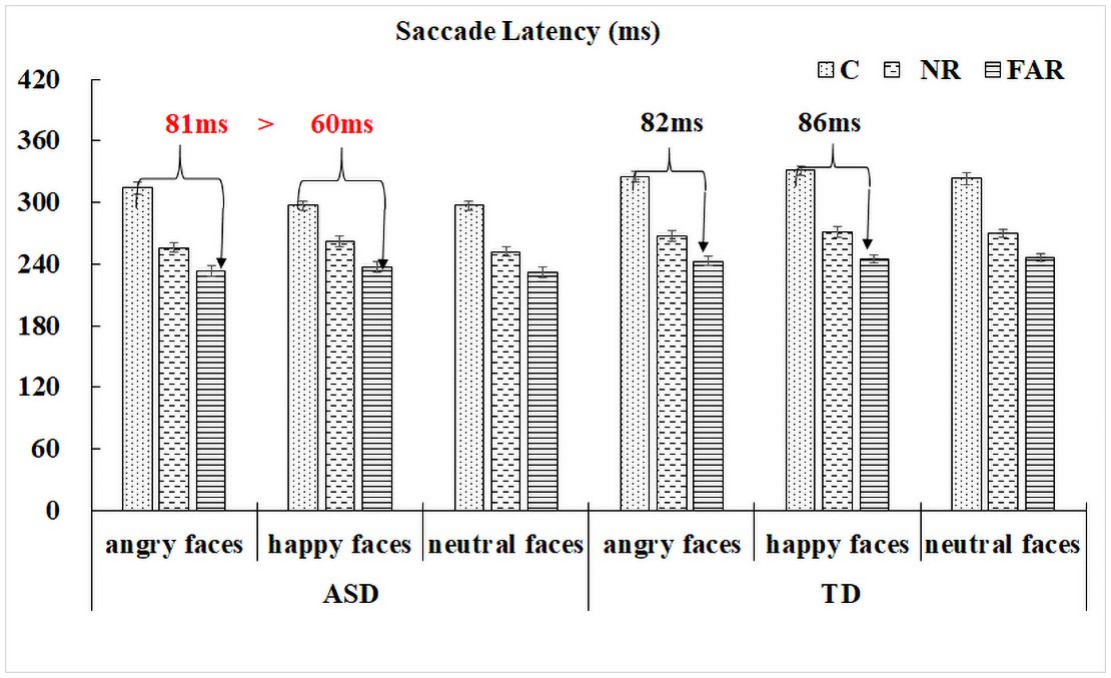
Saccade latency results for each distractor position condition for all distractor types and groups, showing an interaction among three factors in which greater RDE effect amplitude between C and FAR conditions in angry versus happy face distractor condition was observed in the ASD group, but not in the TD group.

For distractor trials, expected remote distractor effects (RDE) were found in all participants, whereby central distractors produced the longest saccade latencies (*M* = 316ms, *SD* = 109ms), followed by the parafoveal distractor condition (*M* = 264ms, *SD* = 76ms) and the peripheral distractor condition (*M* = 241ms, *SD* = 72ms), |*t|s* > 5.60, *p*s < .001. Neither group nor distractor type effect was significant. However, there was a significant three-way interaction amongst group, distractor type (angry vs happy faces) and distractor position (central vs peripheral location), *t* = -2.25, *p* = .025. Detailed analyses revealed different RDE patterns between angry and happy face distractor conditions in the ASD group, *t* = 2.28, *p* = .023, but not in the TD group, *t* = -0.66, *p* = .51. Further analysis in the ASD group revealed that the RDE effect between central and peripheral distractor conditions was greater for angry faces, *t* = -5.58, *p* < .001, compared to happy faces, *t* = -4.50, *p* < .001 (see Fig 4 for details). No other interaction effects were significant (see S2 Table for detailed statistical estimates of the fixed effects for saccade latency).

### Disengagement failure rate

This measure (or DFR) calculated the proportion of trials in which participants failed to disengage from distractors in the first saccade in the central distractor condition. S3 Table illustrates the statistical details of the fixed effects for DFR.

**Fig 5:**
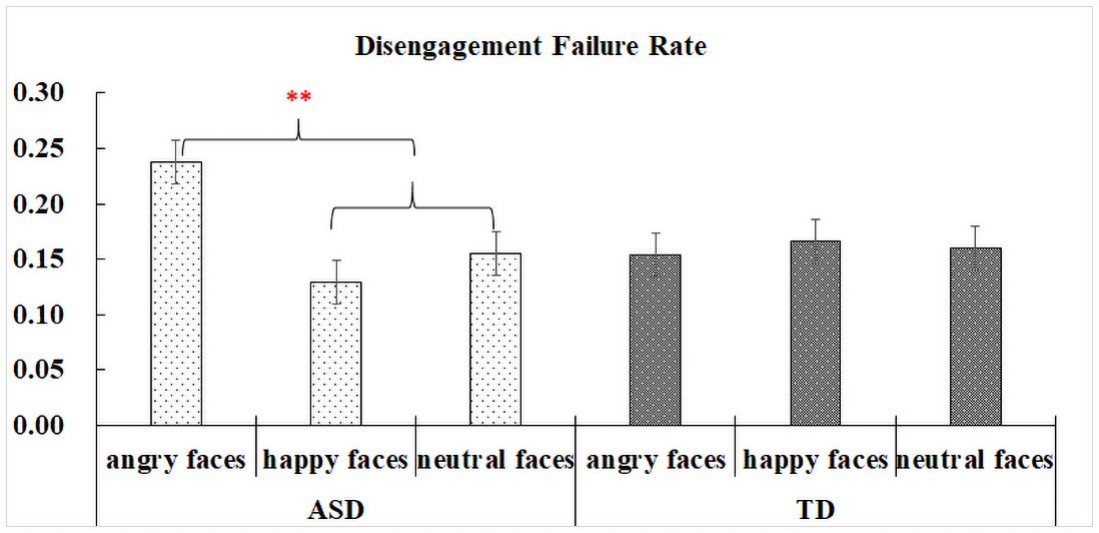
Interactions between group and distractor type on disengagement failure rate.

No overall group difference was found, but a significant distractor type effect showed that DFR was higher in the angry (*M* = 0.19, *SD* = 0.39) face distractor condition compared to happy (*M* = 0.15, *SD* = 0.36) and neutral (*M* = 0.16, *SD* = 0.36) face distractor conditions, |*z*|s > 2.3, *ps* < .05. More importantly, these effects were modulated by group, |*z*|s > 2.3, *ps* < .05, in which higher proportions of DFR in the angry condition versus the other two conditions were significant in the ASD group, |*z*|s > 3, *ps* < .01, but not in the TD group, |*z*|s < 0.5, *ps* >.6 (See Fig 5).

## Discussion

The current study aimed to utilize the Remote Distractor Paradigm to investigate how both the reflexive (exogenous) and voluntary (endogenous) attentional mechanisms are related to the ability to ignore emotional face distractors in children with and without ASD. Consistent with our predictions, the results showed that both the ASD and TD groups made more erroneous saccades towards emotional face distractors, rather than the target, in contrast to neutral face distractors, and no group difference was detected at this reflexive orienting level. At the voluntary attention level the ASD children showed a greater interference from centrally presented angry faces relative to happy or neutral faces, and this finding was observed for both the DFR and saccade latency measures. Together these findings point to greater difficulties in voluntary disengagement from fixated angry faces in the ASD group.

The error rate results show preferential attentional orientation to emotional faces at the involuntary level in both groups. Furthermore, this attentional bias is not associated either with the arousal or with the brightness properties of emotional faces, as the relationships between these properties and error rates were not significant in all participants, *r*s < 0.27, *p*s >.06. Thus, it is the expression that makes the emotional face distractors more attractive in capturing visual attention involuntarily. In addition, this attentional bias to orient to extrafoveal emotional faces could suggest a preserved advantage of processing emotional stimuli pre-attentively in both groups. Importantly, the current error results, which suggest typical reflexive orienting to emotional stimuli in ASD, are consistent with the our previous RDP findings of similar error patterns for non-social distractors in both ASD and typical children [29]. This typical reflexive orienting for emotional faces supports the recent perspectives that social orientation may not be impaired in ASD [40–43], at least at the reflexive level.

Compared to previous studies [16, 23–25] which find typical or faster disengagement from emotional faces in ASD using the SCP paradigm, the current study, using the RDP paradigm provides evidence for disengagement difficulties from angry faces in this population on two different voluntary attention level measures. Firstly, it either takes longer (saccade latency) or, secondly, more saccades (DFR) are needed for ASD children to shift their eyes from the centrally presented angry faces compared to happy or neutral faces. Supportive evidence has also been reported in previous studies with the finding of delayed responses to targets caused by visually frightening distractors [27] and the finding of an increased covert attention to threatening scenes presented for a long time (1250ms) [44]. As an extension to this, the current study itself directly reveals a visual disengagement difficulty for central angry faces at the endogenous attention level in children with ASD. Furthermore, considering that angry faces convey obvious threatening information, this delay could reflect hypervigilance for threats when they are presented centrally in this group [45], and this hypervigilance could result in less flexible attentional disengagement from this type of stimuli in ASD children.

Based on previous reports of a very high prevalence rate of anxious syndromes in ASD, to be at 40%-50% [46, 47], studies have investigated whether atypical attentional disengagement from negative emotion in ASD might be related to the severity of anxiety symptoms, but to date, no significant relationship has been reported. However, those non-significant findings could actually be attributed to the inefficiency of the SCP to differentiate between different levels of attentional processing for emotional information. Findings from the current study suggest that this issue should be explored further to investigate the influence of anxious traits on reflexive orienting and voluntary disengagement from negative emotional stimuli in ASD.

Flexible disengagement has an adaptive relevance in overall development, and also plays a key role in self-regulation of arousal, sensory input, and emotion [48]. For example, attentional disengagement, in order to shift attention, has been taken as an important strategy in the alleviation of discomfort, by diverting the attentional focus from adverse situations in early infancy [49]. Efficient attentional orienting and shifting systems relate to positive emotion regulation in infants [50, 51]. The significance of the voluntary attentional system with respect to novelty detection and processing has also been demonstrated in development [52], and a delayed disengagement can result in either a failed, or a slowed, response to some important social cues in ASD [29, 51]. Slower disengagement from negative stimuli in ASD, based on the findings in the current study, has the potential to delay the detection and processing of other important social stimuli in the environment, and this behavior would have the effect of disrupting the normal flow in communication in ASD compared to TD individuals.

In conclusion, the current findings suggest that children with ASD involuntarily orient to emotional faces, but they have difficulties in disengaging from centrally fixated angry faces at the voluntary level. Inflexible voluntary disengagement from fixated threatening information in ASD could reflect an atypical emotional regulation strategy. An important consequence of this would be the impact upon typical development of higher-level social and communicative functions in ASD.

## Supporting information

S1 Table Fixed effect estimates for error rate. (DOCX)

S2 Table Fixed effect estimates for saccade latency. (DOCX)

S3 Table Fixed effect estimates for DFR. (DOCX)
