# Supplementary material for: The influence of emotional face distractors on attentional orienting in Chinese children with autism spectrum disorder
Source: PLoS One. 2021 May 4;16(5):e0250998. doi: 10.1371/journal.pone.0250998 (PMC8096071; doi:10.1371/journal.pone.0250998)
Supplement: S1 Table — (DOCX) [file pone.0250998.s001.docx]

S1 Table. Fixed effect estimates for error rate

| Effects | Error rate | | |
| --- | --- | --- | --- |
|  | *b* | *SE* | *Z* |
| Group: ASD vs. TD | -0.35 | 0.27 | -1.31 |
| Distractor position: NR vs FAR | -0.02 | 0.05 | -0.33 |
| Distractor type: A vs H | -0.09 | 0.09 | -1.02 |
| Distractor type: A vs N | -0.45 | 0.09 | -4.94*** |
| Distractor type: H vs N | -0.36 | 0.09 | -3.93*** |
| ASD vs. TD x NR vs FAR | 0.06 | 0.11 | 0.59 |
| ASD vs. TD x A vs H | -0.00 | 0.13 | -0.02 |
| ASD vs. TD x A vs N | -0.03 | 0.13 | -0.24 |
| ASD vs. TD x H vs N | -0.03 | 0.13 | -0.23 |
| NR vs FAR x A vs H | -0.16 | 0.13 | -1.23 |
| NR vs FAR x A vs N | -0.32 | 0.13 | -2.43* |
| NR vs FAR x H vs N | -0.16 | 0.13 | -1.21 |
| ASD vs. TD x NR vs FAR x A vs H | -0.03 | 0.27 | -0.12 |
| ASD vs. TD x NR vs FAR x A vs N | -0.34 | 0.27 | -1.28 |
| ASD vs. TD x NR vs FAR x H vs N | -0.31 | 0.27 | -1.17 |

*Note*: **p*<.05, **p*<.01, ****p*<.001

A refers to the angry face distractor condition; H to the happy face distractor condition and N to the neutral face distractor condition.
