# Supplementary material for: The influence of emotional face distractors on attentional orienting in Chinese children with autism spectrum disorder
Source: PLoS One. 2021 May 4;16(5):e0250998. doi: 10.1371/journal.pone.0250998 (PMC8096071; doi:10.1371/journal.pone.0250998)
Supplement: S2 Table — (DOCX) [file pone.0250998.s002.docx]

S2 Table. Fixed effect estimates for saccade latency

| Effects | Saccade latency | | |
| --- | --- | --- | --- |
|  | *b* | *SE* | *t* |
| Group: ASD vs. TD | 0.05 | 0.07 | 0.72 |
| Distractor position: C vs NR | -0.20 | 0.03 | -5.78*** |
| Distractor position: C vs FAR | -0.29 | 0.03 | -8.43*** |
| Distractor position: NR vs FAR | -0.09 | 0.02 | -5.64*** |
| Distractor type: A vs H | 0.01 | 0.02 | 0.35 |
| Distractor type: A vs N | -0.00 | 0.02 | -0.03 |
| Distractor type: H vs N | -0.01 | 0.02 | -0.41 |
| ASD vs. TD x C vs NR | 0.03 | 0.07 | 0.43 |
| ASD vs. TD x C vs FAR | -0.00 | 0.07 | -0.01 |
| ASD vs. TD x NR vs FAR | -0.03 | 0.03 | -0.93 |
| ASD vs. TD x A vs H | -0.02 | 0.04 | -0.56 |
| ASD vs. TD x A vs N | -0.03 | 0.04 | -0.62 |
| ASD vs. TD x H vs N | -0.01 | 0.03 | -0.24 |
| C vs NR x A vs H | 0.03 | 0.02 | 1.47 |
| C vs NR x A vs N | 0.02 | 0.02 | 1.28 |
| C vs NR x H vs N | -0.00 | 0.02 | -0.23 |
| C vs FAR x A vs H | 0.03 | 0.02 | 1.41 |
| C vs FAR x A vs N | 0.03 | 0.02 | 1.44 |
| C vs FAR x H vs N | -0.00 | 0.02 | -0.00 |
| NR vs FAR x A vs H | -0.00 | 0.02 | -0.07 |
| NR vs FAR x A vs N | 0.00 | 0.02 | 0.14 |
| NR vs FAR x H vs N | 0.00 | 0.02 | 0.21 |
| ASD vs. TD x C vs NR x A vs H | -0.07 | 0.04 | -1.72 |
| ASD vs. TD x C vs NR x A vs N | -0.03 | 0.04 | -0.88 |
| ASD vs. TD x C vs NR x H vs N | 0.03 | 0.04 | 0.91 |
| ASD vs. TD x C vs FAR x A vs H | -0.09 | 0.04 | -2.25* |
| ASD vs. TD x C vs FAR x A vs N | -0.06 | 0.04 | -1.50 |
| ASD vs. TD x C vs FAR x H vs N | 0.03 | 0.04 | 0.80 |
| ASD vs. TD x NR vs FAR x A vs H | -0.02 | 0.04 | -0.45 |
| ASD vs. TD x NR vs FAR x A vs N | -0.02 | 0.04 | -0.56 |
| ASD vs. TD x NR vs FAR x H vs N | -0.00 | 0.04 | -0.10 |

*Note*: **p*<.05, **p*<.01, ****p*<.001

A refers to the angry face distractor condition; H to the happy face distractor condition and N to the neutral face distractor condition.
