# Supplementary material for: The influence of emotional face distractors on attentional orienting in Chinese children with autism spectrum disorder
Source: PLoS One. 2021 May 4;16(5):e0250998. doi: 10.1371/journal.pone.0250998 (PMC8096071; doi:10.1371/journal.pone.0250998)
Supplement: S3 Table — (DOCX) [file pone.0250998.s003.docx]

S3 Table. Fixed effect estimates for DFR

| Effects | DFR | | |
| --- | --- | --- | --- |
|  | *b* | *SE* | *Z* |
| Group: ASD vs. TD | -0.26 | 0.46 | -0.58 |
| Distractor type: A vs H | -0.37 | 0.14 | -2.64** |
| Distractor type: A vs N | -0.33 | 0.14 | -2.40* |
| Distractor type: H vs N | 0.04 | 0.14 | 0.29 |
| ASD vs. TD x A vs H | 0.89 | 0.27 | 3.34*** |
| ASD vs. TD x A vs N | 0.62 | 0.26 | 2.39* |
| ASD vs. TD x H vs N | -0.27 | 0.27 | -0.98 |

*Note*: **p*<.05, **p*<.01, ****p*<.001

A refers to the angry face distractor condition; H to the happy face distractor condition and N to the neutral face distractor condition.
